# Effects of high-grain diet feeding on fatty acid profiles in milk, blood, muscle, and adipose tissue, and transcriptional expression of lipid-related genes in muscle and adipose tissue of dairy cows

**DOI:** 10.1186/s40104-023-00847-y

**Published:** 2023-04-08

**Authors:** Qiaorong Cui, Limei Lin, Zheng Lai, Shengyong Mao

**Affiliations:** 1 Jiangsu Key Laboratory of Gastrointestinal Nutrition and Animal Health, Laboratory of Gastrointestinal Microbiology, College of Animal Science and Technology, Nanjing Agricultural University, Nanjing, Jiangsu Province 210095 China; 2 National Center for International Research on Animal Gut Nutrition, Nanjing Agricultural University, Nanjing, 210095 China; 3 National Experimental Teaching Demonstration Center of Animal Science, Nanjing Agricultural University, Nanjing, 210095 China

**Keywords:** Adipose tissue, Fatty acid composition, High-grain diets, Lipid transcriptional profiles, Muscle tissue

## Abstract

**Background:**

High-grain (HG) diets affect lipid metabolism in the liver and mammary tissue of dairy cows, but its effects on muscle and adipose tissue have not been wide evaluated. Thus, the aim of this study is to clarify this issue.

**Methods:**

Twelve Holstein cows were randomly divided into two groups: conventional diet group (CON, *n* = 6) and the HG diet group (*n* = 6). On day 7 of week 4, rumen fluid was sampled to measure pH, milk was sampled to measure components, and blood was sampled to measure biochemical parameters and fatty acid composition. After the experiment, cows were slaughtered to collect muscle and adipose tissue for fatty acid composition and transcriptome analysis.

**Results:**

HG feeding decreased the ruminal pH, milk’s fat content and long-chain fatty acid proportion (*P* < 0.05) and increased the proportion of short- and medium-chain fatty acids in the milk (*P* < 0.05) as compared with CON diets. The concentrations of blood cholesterol, low-density lipoprotein, and polyunsaturated fatty acids in the HG cows were lower than those in CON cows (*P* < 0.05). In muscle tissue, HG feeding tended to increase the triacylglycerol (TG) concentration (*P* < 0.10). Transcriptome analysis revealed changes in the biosynthesis of the unsaturated fatty acids pathway, the regulation of lipolysis in the adipocytes pathway, and the PPAR signalling pathway. In adipose tissue, HG feeding increased the concentration of TG and decreased the concentration of C18:1 *cis*9 (*P* < 0.05). At the transcriptome level, the fatty acid biosynthesis pathway, linoleic acid metabolism pathway, and PPAR signalling pathway were activated.

**Conclusion:**

HG feeding leads to subacute rumen acidosis and a decreased milk fat content. The fatty acid profiles in the milk and plasma of dairy cows were changed by HG feeding. In muscle and adipose tissue, HG feeding increased TG concentration and up-regulated the expression of genes related to adipogenesis, while down-regulated the expression of genes related to lipid transport. These results complement our knowledge of the fatty acid composition of muscle and adipose tissue in dairy cows and expand our understanding of the mechanisms by which HG diets affect lipid metabolism in muscle and adipose tissue.

**Supplementary Information:**

The online version contains supplementary material available at 10.1186/s40104-023-00847-y.

## Background

Diets rich in grain, often used for maintaining elevated energy levels in lactating cows, may be responsible for the occurrence of subacute rumen acidosis (SARA), as pointed out by several authors [1, 2]. SARA represents a disorder of the rumen and features intermittent and moderate drops in rumen pH [3]. High-grain (HG) diets containing excess non-structural carbohydrates and insufficient crude fibre allow the production of ruminal volatile fatty acids faster than their buffer, thereby reducing rumen pH and contributing to SARA development [2, 4]. SARA is a widespread problem in mid-lactation cows and causes serious economic losses. Field studies in Wisconsin revealed that the prevalence of SARA in mid-lactating cows was 26% [5], and the economic cost associated with SARA per cow is about $400 annually [6]. Grain-based SARA challenges are often accompanied by a number of typical complications, including reduced feed intake, milk fat depression, laminitis, and liver abscesses [7, 8].

Milk fat is one of the principal nutritional components of milk. Fatty acids in milk originate from the de novo synthesis of mammary tissue and uptake from blood [9]. The types and amounts of fatty acids taken from blood by the mammary tissue are affected by many factors, such as diet, the rumen fermentation pattern, and the lipid metabolism status of other tissues [8, 10, 11]. Blood serves as a lipid carrier between the mammary tissue and other tissues, thus assisting in milk fat synthesis. Triacylglycerol (TG) and cholesterol are the core components of blood lipids, which are transported by lipoproteins [12]. Numerous studies have demonstrated that HG diets change the lipid profiles of milk and blood [13, 14], possibly by affecting the production of the milk fat precursor acetic acid, the expression of mammary gland fat synthesis genes, and the redistribution of liver fat [15, 16]. Although muscle and adipose tissue play an indispensable role in the lipid metabolism of lactating ruminants, there is a lack of studies on the effects of grain-based SARA on the fatty acid profiles of muscle and adipose tissue.

Muscle is a major contributor to the body’s ability to adapt to physiological changes and maintain metabolic homeostasis during lactation [17]. In early lactation cows, the lipid metabolism of muscle is enhanced to relieve the pressure on liver lipid metabolism [18]. Adipose tissue acts as a fatty acid logistics warehouse in animals, capable of storing fatty acids in the form of triglycerides during periods of energy excess or mobilising adipocytes to release fatty acids during periods of energy deficit [19]. In early lactation, dairy cows may mobilize about 1 kg of adipose tissue fat per day to help produce milk fat [20].

In the animal body, the fat content in tissues is not only affected by the substrate, but also affected by the expression of related genes involved in lipid metabolism [21]. Studies in beef cattle have shown that feeding HG diets can affect fat deposition by altering the expression of genes related to fat metabolism in muscle and adipose tissue, such as *SCD*, *FABP4*, and *ACACA* [22, 23]. So far, a large number of studies have revealed the effect of HG diet-induced SARA on the expression of lipid metabolism-related genes in liver and mammary gland tissues of dairy cows [24, 25], but the effect on the expression of lipid metabolism-related genes in muscle and adipose tissue has not been extensively studied.

Therefore, the objective of this study was to determine the effects of HG feeding on the fatty acid profiles of milk and blood and on the lipid metabolism in the muscle and adipose tissue of dairy cows. We hypothesised that HG feeding alters the fatty acid profiles of milk, blood, muscle, and adipose tissue and affects the transcriptional expression of lipid-related genes in the muscle and adipose tissue of dairy cows.

## Materials and methods

### Animals and experimental treatments

Twelve healthy multiparous late-lactation Holstein cows (BW = 613 ± 58 kg, milk yield = 18.2 ± 2.7 kg/d, DIM = 233 ± 23 (mean ± SD)) with rumen fistulas were chosen. The cows were randomly divided into two groups of six: the conventional diet group (CON) and the HG diet group. The experiment lasted for 4 weeks, including 1 week for pre-feeding and 3 weeks for the trial period. During the pre-feeding period, both groups were fed a conventional diet (forage-to-concentrate 6:4, dry matter (DM) basis, Table 1). However, in the feeding trial, the cows in the CON group continued to receive a conventional diet, while the HG cows were switched to a HG diet (forage-to-concentrate 4:6, dry matter (DM) basis, Table 1). The dietary concentrate level of the HG group was increased gradually during the first 2 d (by approximately 10% each day, based on the CON diet). The diets had comparable CP and were formulated according to NRC (2001) to meet the energy and milk production requirements of a 620-kg Holstein cow yielding 25 kg/d of milk with 4.0% milk fat and 3.5% milk protein [27]. All cows were fed twice a day at 08:00 and 20:00 (5%–10% orts on an as-fed basis were allowed) and milked twice before feeding. All cows were healthy and had free access to fresh water throughout the experiment.

**Table 1:** Ingredients and nutritional composition of the conventional diet (CON) and the high-grain diet (HG)

Item	Diet	
	CON	HG
Ingredient, % of DM		
Alfalfa hay	24.00	17.00
Oaten hay	24.00	17.00
Corn silage	12.00	6.00
Corn grain	19.40	24.92
Soybean	13.50	13.48
Barley	—	12.00
DDGS^1^	3.80	5.91
CaCO_3_	0.80	1.48
Ca(HCO_3_)_2_	1.10	0.92
NaCl	0.40	0.37
Premix^2^	1.00	0.92
Nutrient composition^3^		
DM, %	46.77	48.03
CP, % of DM	16.16	16.12
Crude fat, % of DM	3.05	3.05
NDF, % of DM	36.14	29.92
NFC^4^, % of DM	35.39	42.34
Starch, % of DM	17.96	27.82
Ash, % of DM	5.97	4.87
Ca, % of DM	1.14	1.18
P, % of DM	0.52	0.51
NE_L_^5^, Mcal/kg of DM	1.57	1.64
NFC/NDF	0.97	1.42

^1^Dried distillers grains with solubles

^2^Premix contained the following ingredients per kilogram of diet: vitamin A, 22.5 kIU; vitamin D_3_, 5.0 kIU; vitamin E, 37.5 IU; vitamin K_3_, 5.0 mg; Mn, 63.5 mg; Zn, 111.9 mg; Cu, 25.6 mg; and Fe, 159.3 mg

^3^*DM* dry matter, *CP* crude protein, *NDF* neutral detergent fiber, *NFC* non-fibrous carbohydrate, *NE* net energy

^4^Calculated as NFC = 100 − (%NDF + %CP + %ether extract + %ash). NDF was inclusive of residual ash

^5^Calculated based on Ministry of P. R. China recommendations [26]

### Ruminal and diet sample collection and analysis

Rumen contents were collected through rumen fistulas at 0, 2, 4, 6, 8, and 12 h after the morning feeding on d 6 and 7 of week 4. The samples were immediately filtered using four layers of sterile cheesecloth, and the rumen pH was measured using a portable pH metre (HI 99161; Hanna, Woonsocket, RI, USA).

The feed offered and orts were measured and recorded daily to calculate feed intake. Fresh feed and orts were sampled daily and stored at −20 °C until required for feed DM analysis by oven drying at 55 °C for 72 h. The analytical DM content was determined by drying at 135 °C for 3 h [28].

### Blood and milk sampling and analysis

At 4 h after the morning feeding on d 7 of week 4, blood samples were collected from the tail vein into vacutainer tubes using heparin sodium as an anticoagulant. Plasma was separated by centrifuging the blood samples (10 min, 3000 × *g* at 4 °C) and immediately storing them at −20 °C until required for further analysis. Plasma biochemical parameters, such as glucose (Glu), total protein (TP), total cholesterol (TCHO), TG, total bile acid (TBA), high-density lipoprotein (HDL), and low-density lipoprotein (LDL), were determined using an automatic biochemical analyser (Beckman AU5800, Beckman Coulter, Brea, CA, USA). Another portion of the plasma was stored at −20 °C to be used for fatty acid composition analysis.

Milk production was recorded during the experiment. On d 6 and 7 of week 4, milk samples were collected during 2 consecutive milkings per day. The samples on the same day were mixed to analyse milk composition by mid-infrared spectroscopy (Fossomatic 4000, Foss Electric A/S, Hillerød, Denmark). The rest milk in d 7 of week 4 was stored at −20 °C until needed for fatty acid composition analysis.

### Slaughter and tissue sample collection

At the end of the experimental period, the cows were humanely euthanised using a high-tension current stunning method. After slaughter, samples of the *longissimus dorsi* muscle and abdominal adipose tissue were collected from each cow. The samples were stored in liquid nitrogen for TG content, fatty acid composition, transcriptome, and qRT-PCR analysis.

### Triacylglycerol concentration detection

The TG content in muscle and adipose tissue was measured using commercial kits (Nanjing Jiancheng Bioengineering Institute, Jiangsu, China) following the manufacturer’s instructions.

### Fatty acid composition analysis

The extraction of milk fat and the preparation of fatty acid methyl esters (FAMEs) were performed according to the procedure published by Nafikov et al. [29]. The extraction of plasma fat and the preparation of FAMEs were performed as described by Xu et al. [30]. The FAMEs were placed into gas chromatography vials and used for chromatographic analysis.

An Agilent 8890 gas chromatograph (Agilent Technologies, Santa Clara, CA, USA) equipped with an auto-sampler, auto-injector, split/splitless injector, and flame ionisation detector was used to analyse the FAMEs using nitrogen gas as a carrier at a constant flow rate of 1.1mL/min. One microlitre of FAMEs in hexane was injected into a fused silica capillary column (Agilent J&W Advanced Capillary GC Columns, Netherlands; 100 m × 0.25 mm i.d., with 0.2 μm film thickness) with the initial column temperature set at 150 °C and held at this temperature for 5 min. Then, the column temperature was increased to 200 °C at a rate of 2 °C/min and held at this temperature for 10 min, with a subsequent increase to 220 °C at a rate of 5 °C/min, where it was held for 35 min [30]. The injector and detector temperatures were set constant at 260 and 280 °C, respectively. The peak area was measured using OpenLab CDS (Agilent Technologies), and the peaks were identified by comparing the retention times with FAME standards (18919-1AMP; Sigma Chemicals, St. Louis, MO, USA). Fatty acid composition analysis of muscle and adipose tissue was performed under the same operating conditions for the plasma.

### Transcriptome sequencing and data analysis

The total RNA of the muscle of 12 dairy cows was extracted using a TRIzol reagent kit (Takara Bio, Otsu, Japan) following the manufacturer’s protocol. The concentration and integrity of the RNA were determined using a NanoDrop spectrophotometer (Thermo, Waltham, MA, USA) and 1.4% agarose formaldehyde gel electrophoresis, respectively. A total of 1 µg of RNA was used to construct a sequencing library using a NEBNext®Ultra™RNA Library Prep Kit (Illumina, San Diego, CA, USA). The prepared library was sequenced using the Illumina HiSeq platform (HiSeqTM 2500). Trimmomatic was utilised to filter out the low-quality reads [31]. The high-quality filtered sequences were mapped to the bovine Bowtie2 (V2.1.0) reference genome using Hisat2 [32], and the coverage of the sequencing results was evaluated in Qualimap (V2.2.1) [33]. The gene expression level was calculated by subread software using the FPKM value [34], while the principal component analysis (PCA) and partial least squares discriminant analysis (PLS-DA) were performed using SIMCA (SIMCA v. 14.1, Umetrics, Umea, Sweden). The DESeq R package (v1.18.0, 2012) was used to screen differentially expressed genes (DEGs) with a fold change ≥ 2 and FDR < 0.05 [35]. A KEGG pathway enrichment analysis of the DEGs was conducted using the R package clusterProfiler (v4.2.2). Transcriptome analysis of adipose tissue was performed under the same operating conditions.

### Quantitative real-time PCR analysis

Total RNA was extracted from 200 mg muscle samples from 12 cows using TRIzol (Invitrogen, Carlsbad, CA, USA). The cDNA was synthesised using PrimeScript RT Master Mix Perfect Real Time (Takara Co., Otsu, Japan). The primers were designed in Primer 5.0 (Premier Biosoft International, Palo Alto, CA, USA) and are presented in Table S1 in Additional file 1. The cDNA samples were amplified using SYBR Green (Takara Co., Otsu, Japan) on the QuantStudio 7Flex rapid real-time PCR system and a 20-µL amplification system containing 10 µL SYBR Premix Ex TaqTM, 0.8 µL primers, 0.4 µL ROX, 2 µL cDNA, and 6.8 µL ddH_2_O. The protocol consisted of keeping the samples at 95 °C for 30 s, 95 °C for 5 s, and 60 °C for 20 s, with 40 cycles. *GAPDH* was used as the reference gene. The relative expression of genes was calculated using the 2^−^^ΔΔCt^ method ((Ct target gene − Ct β-actin) treatment − (Ct target gene − Ct β-actin) control) [36]. The adipose tissue sequencing data were validated using the same method.

### Statistical analysis

A minimum sample size of 5 cows for each group was calculated using power analysis before the start of the experiment. Each group was to have a power of at least 80% given the effect size of 1 with a type I error of 1% using G*Power 3.1.9.6 based on *t*-test of the difference between two dependent means [37]. In order to ensure statistical power > 0.8, we selected 6 cows for each group in the experiment.

The data were analyzed by the SPSS software package (IMB SPSS v. 24, IBM Corp., Armonk, NY, USA). The pH, DMI, and milk yield and components data were analyzed using the linear mixed-effects models (MIXED) procedure. The effects of diet, day, and their interactions were considered fixed factors, and the effect of cows was considered random. Other indicators (fatty acid composition, biochemical indicators, and mRNA expression level) were statistically analyzed by independent *t*-test. We considered that a *P* < 0.05 indicates a significant difference, and a *P* < 0.10 indicates a trend.

## Results

### Rumen pH

**Fig. 1:**
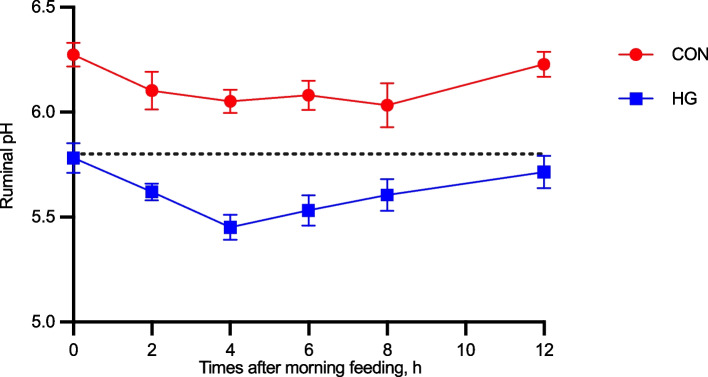
Dynamic variation of ruminal pH between the conventional diet group (CON) and the high-grain diet group (HG). The black dotted line parallel to the *X*-axis represents pH = 5.8, CON = conventional diet group; HG = high-grain diet group. Error bars indicate SEM

The ruminal pH of the HG group significantly decreased (6.1 vs. 5.6, *P* < 0.001) compared with that of the CON group and was lower than that of the CON group at each sampling time point (Fig. 1). The ruminal pH of the cows fed a HG diet was below 5.8 for more than 3 h within a day.

### Milk yield and composition, DMI

As shown in Table 2, the milk fat content was lower (*P* = 0.019) in the HG group compared with the CON group. On the contrary, cows in HG group had a higher content of the milk protein (*P* = 0.049) than CON cows. However, the milk yield (*P* = 0.338), DMI (*P* = 0.436), milk lactose content (*P* = 0.821), total solid content (*P* = 0.292), somatic cell count (*P* = 0.829), and urea nitrogen content (*P* = 0.152) were unaffected by the HG diet.

**Table 2:** Effects of high-grain diet (HG) or conventional diet (CON) on milk yield and composition of dairy cows^1^

Item	CON	HG	*P*-value
Milk yield, kg/d	14.53 ± 2.99	17.91 ± 1.54	0.338
DMI, kg/d	22.19 ± 1.17	23.69 ± 1.43	0.436
Milk fat, %	4.49 ± 0.15	3.92 ± 0.14	0.001
Milk protein, %	3.85 ± 0.17	4.13 ± 0.08	0.049
Milk lactose, %	4.96 ± 0.05	4.94 ± 0.08	0.747
Total solid, %	13.69 ± 0.25	13.34 ± 0.19	0.135
Somatic cell count, ×10 cells/mL	189.50 ± 76.86	212.58 ± 70.25	0.758
Urea nitrogen, mg/dL	17.95 ± 0.34	18.82 ± 0.45	0.105

^1^Data expressed as mean ± SEM, *n* = 6. *DMI* Dry matter intake

### Plasma biochemical parameters

**Fig. 2:**
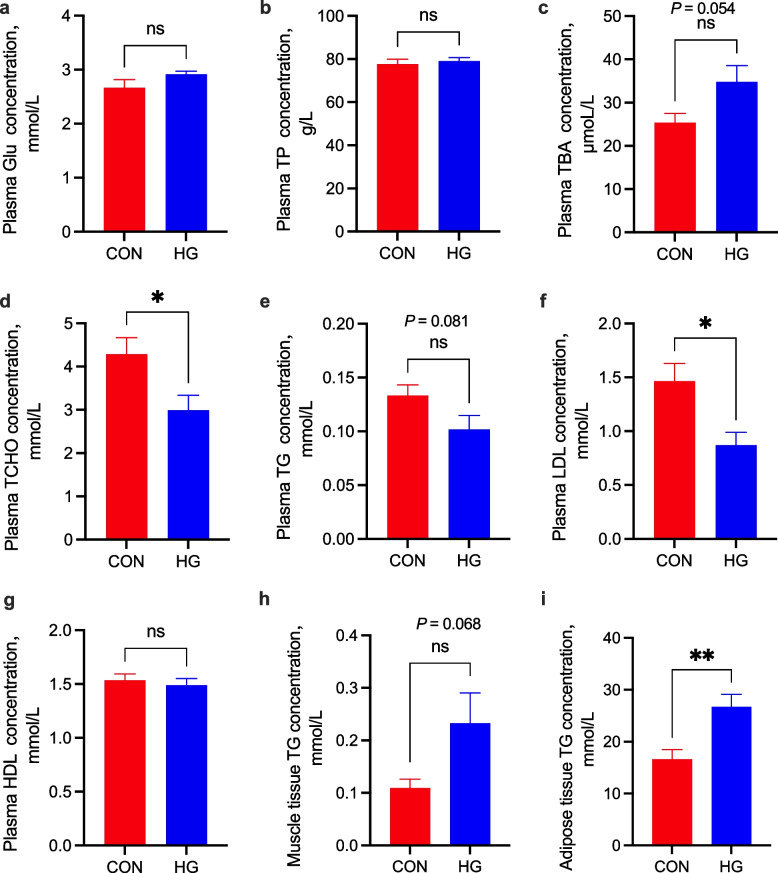
Differences in biochemical indexes related to lipid metabolism between the conventional diet group (CON) and the high-grain diet group (HG). **a** Plasma glucose concentration (Glu); **b** Plasma total protein concentration (TP); **c** Plasma total bile acid concentration (TBA); **d** Plasma total cholesterol concentration (TCHO); **e** Plasma triacylglycerol concentration (TG); **f** Plasma low-density lipoprotein concentration (LDL); **g** Plasma high-density lipoprotein concentration (HDL); **h** Muscle tissue triacylglycerol concentration (TG); **i** Adipose tissue triacylglycerol concentration ns, no significance, ^*^P < 0.05, ^**^P < 0.01

The cows on the HG diet had lower plasma TCHO and LDL concentrations (*P* = 0.031 and *P* = 0.015, respectively) than the cows on the CON diet (Fig. 2d and f, respectively). Furthermore, the HG diet tended to increase the plasma TBA concentration (*P* = 0.054, Fig. 2c) and decrease the plasma TG concentration (*P* = 0.081, Fig. 2e). No significant difference was observed in the concentrations of plasma GLU, TP, and HDL (*P* = 0.143, *P* = 0.650, and *P* = 0.605, respectively) between the two groups (Fig. 2a, b and g, respectively).

### Fatty acid composition of milk, plasma, muscle, and adipose tissue

The effect of HG feeding on the fatty acid composition of milk is shown in Table 3. The majority of fatty acids in dairy cow milk were saturated fatty acids (SFAs) (average 65.68%, SEM = 1.33%) followed by unsaturated fatty acids (UFAs) (average 34.32%, SEM = 1.33%), which were primarily composed of monounsaturated fatty acids (MUFAs) (average 26.67%, SEM = 1.42%). The average proportion of polyunsaturated fatty acids (PUFAs) in milk was 7.65% (SEM = 0.53%). The proportions of milk C12:0 (*P* = 0.013) and C ≤ 16 (*P* = 0.048) in the HG group were higher than those in the CON group, but the proportions of milk C17:0 (*P* = 0.036) and C > 16 (*P* = 0.048) were lower than those in the CON group. In addition, HG feeding tended to increase the proportions of milk C10:0 (*P* = 0.064), C11:0 (*P* = 0.064) and C16:1 *cis*9 (*P* = 0.094), while the opposite trend was observed for the proportion of C18:0 (*P* = 0.091).

**Table 3:** Effects of high-grain diet (HG) or conventional diet (CON) on milk fatty acid proportions^1^

Item	CON	HG	SEM	*P*-value
C4:0	1.13	1.40	0.12	0.263
C6:0	1.26	1.46	0.10	0.354
C8:0	0.84	0.95	0.06	0.433
C10:0	3.13	3.67	0.14	0.064
C11:0	0.74	1.04	0.08	0.064
C12:0	4.33	5.59	0.27	0.013
C13:0	0.60	0.88	0.09	0.132
C14:0	12.92	14.42	0.55	0.186
C14:1 *cis*9	2.22	3.06	0.29	0.150
C15:0	3.15	3.94	0.28	0.185
C16:0	22.29	24.04	0.83	0.315
C16:1 *cis*9	4.02	4.85	0.24	0.094
C17:0	1.79	1.25	0.13	0.036
C17:1 *trans*10	1.25	1.39	0.10	0.546
C18:0	11.67	7.79	1.14	0.091
C18:1 *trans*9	0.50	0.44	0.07	0.663
C18:1 *cis*9	19.90	14.00	1.71	0.105
C18:2 *trans*9,12	0.16	0.23	0.02	0.195
C18:2 *cis*9,12	5.65	7.06	0.44	0.122
C20:0	0.60	0.49	0.06	0.353
C20 *cis*11	0.85	0.87	0.05	0.895
C20:3 *cis*8,11,14	0.35	0.35	0.03	0.983
C20:3 *cis*11,14,17	0.64	0.85	0.07	0.161
SFA	64.45	66.91	1.33	0.387
UFA	35.55	33.09	1.33	0.387
MUFA	28.74	24.60	1.42	0.172
PUFA	6.81	8.49	0.53	0.123
C ≤ 16	56.62	65.29	2.26	0.048
C > 16	43.38	34.71	2.26	0.048

*SEM *Standard error of the mean between the two treatments, *SFA *Saturated fatty acid, *UFA *Unsaturated fatty acid, *MUFA *Monounsaturated fatty acids, *PUFA *Polyunsaturated fatty acids

^1^g/100 g of total fatty acid

The effect of HG feeding on the fatty acid composition of plasma is shown in Table 4. UFAs accounted for the largest proportion of plasma fatty acids (average 60.19%, SEM = 1.29%) and consisted of PUFAs (average 42.46%, SEM = 1.54%) and MUFAs (average 17.73%, SEM = 0.51%). The average proportion of plasma SFAs was 39.82% (SEM = 1.29%). The cows on the HG diet had higher proportions of plasma C4:0 (*P* = 0.029), C6:0 (*P* = 0.020), and C17:1 *trans*10 (*P* = 0.041) but lower proportions of plasma C16:1 *cis*9 (*P* = 0.032), C18:2 *cis*9,12 (*P* = 0.033) and PUFAs (*P* = 0.026) than the cows fed a CON diet. HG feeding showed a tendency for higher proportions of plasma SFAs (*P* = 0.051) and C ≤ 16 (*P* = 0.058), as well as a trend towards lower proportions of plasma C20:3 *cis*8,11,14 (*P* = 0.093), UFAs (*P* = 0.051), and C > 16 (*P* = 0.058).

**Table 4:** Effects of high-grain diet (HG) or conventional diet (CON) on plasma fatty acid proportions^1^

Item	CON	HG	SEM	*P*-value
C4:0	3.16	4.68	0.35	0.029
C6:0	2.81	3.61	0.17	0.020
C8:0	0.73	1.02	0.09	0.125
C10:0	0.49	0.63	0.05	0.201
C11:0	0.41	0.46	0.04	0.566
C12:0	0.64	0.72	0.04	0.293
C13:0	0.57	0.59	0.04	0.852
C14:0	1.00	1.14	0.34	0.848
C14:1 *cis*9	0.55	0.68	0.07	0.377
C15:0	0.66	0.55	0.09	0.573
C16:0	11.32	12.62	0.57	0.279
C16:1 *cis*9	0.89	0.64	0.06	0.032
C17:0	0.70	0.61	0.05	0.375
C17:1 *trans*10	11.16	13.86	0.67	0.041
C18:0	14.31	15.03	0.26	0.181
C18:1 *trans*9	2.48	1.78	0.24	0.169
C18:2 *trans*9,12	0.52	0.58	0.03	0.321
C18:2 *cis*9,12	38.89	33.10	1.40	0.033
C20:0	0.54	0.64	0.05	0.342
C18:3 *cis*9,12,15	0.89	0.92	0.09	0.894
C20 *cis*11	1.81	1.61	0.18	0.598
C20:3 *cis*8,11,14	2.80	2.49	0.09	0.093
C20:3* cis*11,14,17	2.66	2.06	0.19	0.120
SFA	37.34	42.29	1.29	0.051
UFA	62.66	57.71	1.29	0.051
MUFA	16.89	18.57	0.51	0.116
PUFA	45.77	39.14	1.54	0.026
C ≤ 16	23.23	27.33	1.10	0.058
C > 16	76.77	72.67	1.10	0.058

*SEM *Standard error of the mean between the two treatments, *SFA *Saturated fatty acid, *UFA* Unsaturated fatty acid, *MUFA* Monounsaturated fatty acids, *PUFA* Polyunsaturated fatty acids

^1^g/100 g of total fatty acid

The effect of HG feeding on the fatty acid composition of the muscle is shown in Table 5. UFAs had the largest proportion of fatty acids in the muscle tissue of dairy cows (average 67.11%, SEM = 1.01%) and were primarily composed of MUFAs (average 40.45%, SEM = 1.51%). The average proportions of PUFAs and SFAs in muscle tissue were 26.66% (SEM = 1.94%) and 32.89% (SEM = 1.01%), respectively. Furthermore, different diets did not influence the fatty acid proportions in muscle.

**Table 5:** Effects of high-grain diet (HG) or conventional diet (CON) on muscle fatty acid proportions^1^

Item	CON	HG	SEM	*P*-value
C14:0	1.46	1.54	0.11	0.731
C14:1 *cis*9	0.99	1.11	0.10	0.575
C15:0	0.46	0.52	0.04	0.442
C16:0	14.89	14.48	0.58	0.744
C16:1 *cis*9	5.51	6.00	0.49	0.633
C17:0	1.41	1.50	0.07	0.528
C17:1 *trans*10	2.01	1.64	0.28	0.542
C18:0	15.27	14.27	0.55	0.388
C18:1 *trans*9	1.20	1.35	0.11	0.539
C18:1 *cis*9	29.28	29.49	1.05	0.927
C18:2 *trans*9,12	1.49	3.41	1.23	0.473
C18:2 *cis*9,12	17.76	16.02	2.05	0.695
C20:1 *cis*11	1.19	1.14	0.07	0.737
C20:3 *cis*8,11,14	1.71	2.26	0.22	0.249
C20:3 *cis*11,14,17	5.38	5.29	0.38	0.908
SFA	33.48	32.30	1.01	0.586
UFA	66.52	67.70	1.01	0.586
MUFA	40.18	40.72	1.51	0.868
PUFA	26.34	26.98	1.94	0.878
C ≤ 16	23.30	23.65	0.93	0.864
C > 16	76.70	76.35	0.93	0.864

*SEM  *Standard error of the mean between the two treatments, *SFA *Saturated fatty acid, *UFA *Unsaturated fatty acid, *MUFA *Monounsaturated fatty acids, *PUFA *Polyunsaturated fatty acids

^1^g/100 g of total fatty acid

The effect of HG feeding on the fatty acid composition of adipose tissue is shown in Table 6. UFAs were the main component of fatty acids in the adipose tissue of dairy cows (average 78.23%, SEM = 3.79%) and were mainly composed of MUFAs (average 49.76%, SEM = 3.66%). The average proportions of PUFAs and SFAs in muscle tissue were 28.47% (SEM = 1.20%) and 21.77% (SEM = 3.79%), respectively. The proportion of C18:1 *cis*9 (*P* = 0.013) in the HG group was lower than in the CON group. Moreover, HG feeding tended to increase the proportions of C16:1 *cis*9 (*P* = 0.055) in adipose tissue.

**Table 6:** Effects of high-grain diet (HG) or conventional diet (CON) on adipose tissue fatty acid proportions^1^

Item	CON	HG	SEM	*P*-value
C14:0	14.38	6.70	3.11	0.233
C14:1 *cis*9	9.44	16.52	2.99	0.254
C15:0	3.30	3.11	0.14	0.523
C16:1 *cis*9	21.11	27.54	1.69	0.055
C17:0	9.64	6.41	1.68	0.363
C17:1 *trans*10	4.02	4.67	0.26	0.221
C18:1 *cis*9	7.00	4.31	0.59	0.013
C18:2 *trans*9,12	3.16	3.07	0.15	0.772
C18:2 *cis*9,12	20.93	20.51	1.05	0.853
C18:3 *cis*6,9,12	2.96	3.34	0.18	0.316
C20 *cis*11	2.64	2.27	0.13	0.188
C20:3 *cis*8,11,14	1.41	1.54	0.12	0.597
SFA	27.33	16.22	3.79	0.152
UFA	72.67	83.78	3.79	0.152
MUFA	44.21	55.31	3.66	0.135
PUFA	28.46	28.47	1.20	0.999
C ≤ 16	48.24	53.87	2.24	0.231
C > 16	51.76	46.13	2.24	0.231

*SEM *Standard error of the mean between the two treatments, *SFA* Saturated fatty acid, *UFA* Unsaturated fatty acid, *MUFA* Monounsaturated fatty acids, *PUFA* Polyunsaturated fatty acids

^1^g/100 g of total fatty acid

### Triacylglycerol concentration of muscle and adipose tissue

HG feeding led to an increase in TG concentration in muscle (*P* = 0.068) compared with the CON group (Fig. 2h). In addition, the TG concentration in the adipose tissue (*P* = 0.007) of the HG group was markedly higher than that in the CON group (Fig. 2i).

### Transcriptome profile analysis of the muscle tissue

**Fig. 3:**
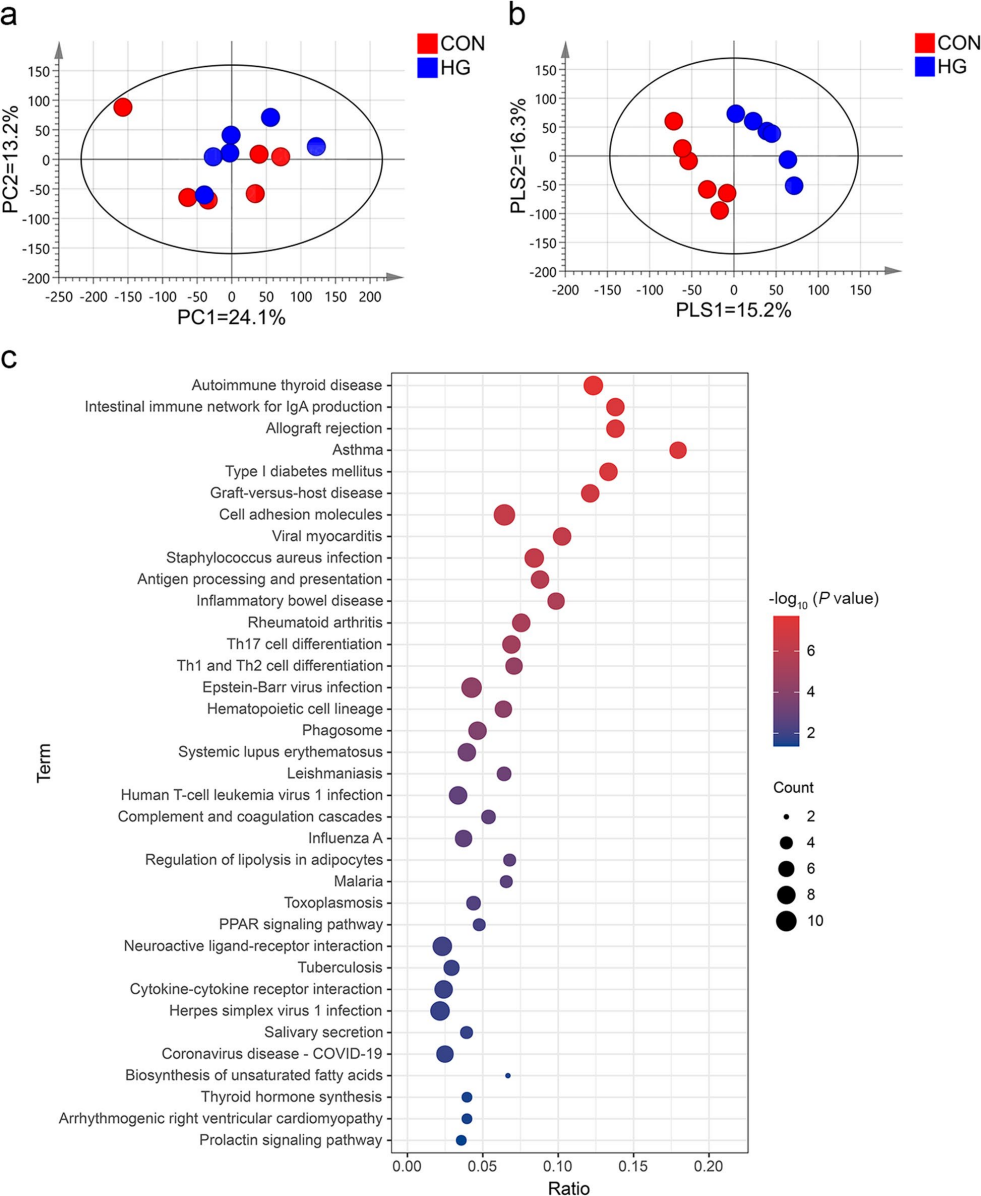
The principal component analysis (PCA) and the partial least squares discriminant analysis (PLS-DA) of the muscle tissue transcriptome for dairy cows in the conventional diet group (CON; 40% concentrate, *n* = 6) group and the high-grain diet group (HG; 60% concentrate, *n* = 6) group. **a** PCA score scatter plot; **b** PLS-DA score scatter plot. **c** Kyoto Encyclopedia of KEGG pathway enrichment analysis of DEGs. The *X*-axis and *Y*-axis in (**c**) represent the pathway and the pathway enrichment ratio, respectively

PCA was performed on the gene expression profiles to study the clustering of samples. The PCA plot (Fig. 3a) shows that HG and CON gene expression profiles in muscle tissue did not seem to form obvious clustering. But the first PC (PC1) explained 24.1% of the variance between samples, while the second PC (PC2) was responsible for 13.2% of the variation. Likewise, axes 1 and 2 of PLS-DA explained 15.2% and 16.3% of the total variation, respectively (Fig. 3b). A total of 218 DEGs; fold change ≥ 2 and FDR < 0.05 were identified from the 22,586 genes in the muscle transcriptome data, including 121 up-regulated genes and 97 down-regulated genes in the HG group. The pathway analysis of DEGs was conducted using the clusterProfiler R package (v4.2.2) according to the KEGG pathway database. A total of 36 significantly changed pathways were identified, as shown in Fig. 3c (*P* < 0.05). Among them, three pathways are related to lipid metabolism: the regulation of lipolysis in adipocytes, the biosynthesis of UFAs, and the PPAR signalling pathway. Compared with the CON group, a total of eight DEGs were identified among these pathways in the HG group, of which two genes were significantly up-regulated and six genes were significantly down-regulated (Table 7). In the regulation of lipolysis in the adipocytes pathway, *CGA* expression in the muscle tissue of the cows fed a HG diet was up-regulated, while the gene expressions of *FABP4*, *PTGER3*, and *CIDEC* were down-regulated (Fig. S1 in Additional file 1). In the biosynthesis of the UFA pathway, HG feeding down-regulated the gene expressions of *SCD5* and *ELOVL6* in the muscle tissue (Fig. S2 in Additional file 1). In the PPAR signalling pathway, the gene expressions of *FABP4*, *ADIPOQ*, and *SCD5* in muscle tissue were down-regulated by HG feeding, while the gene expression of *APOA2* were up-regulated (Fig. S3 in Additional file 1).

**Table 7:** Differentially expressed genes in muscle tissue due to high-grain diet feeding^1^

Pathway	Gene symbol^2^	FDR	Log_2_FC	Gene-ID
Regulation of lipolysis in adipocytes pathway	*CGA*	< 0.001	1.438	12,373
	*FABP4*	< 0.001	–1.391	18,034
	*PTGER3*	< 0.001	–1.822	4070
	*CIDEC*	< 0.001	–1.066	27,073
Biosynthesis of unsaturated fatty acids	*SCD5*	< 0.001	–1.005	8601
	*ELOVL6*	< 0.001	–1.001	7957
PPAR signalling pathway	*APOA2*	< 0.001	2.225	2917
	*FABP4*	< 0.001	–1.391	18,034
	*ADIPOQ*	< 0.001	–1.602	622
	*SCD5*	< 0.001	–1.005	8601

^1^Results based on RNA sequencing, *n* = 6. The gene expressions were considered to be significantly altered when FC ≥ 2 and FDR < 0.05

^2^*CGA* Glycoprotein hormones alpha chain precursor, *FABP4* Fatty acid-binding protein 4, *PTGER3* Prostaglandin E2 receptor EP3 subtype, *CIDEC* Cell death activator, *SCD5* Stearoyl-CoA desaturase 5, *ELOVL6* Elongation of very long chain fatty acids protein 6, *ADIPOQ* Adiponectin, *APOA2* Apolipoprotein A-2

### Transcriptome profile analysis of adipose tissue

**Fig. 4:**
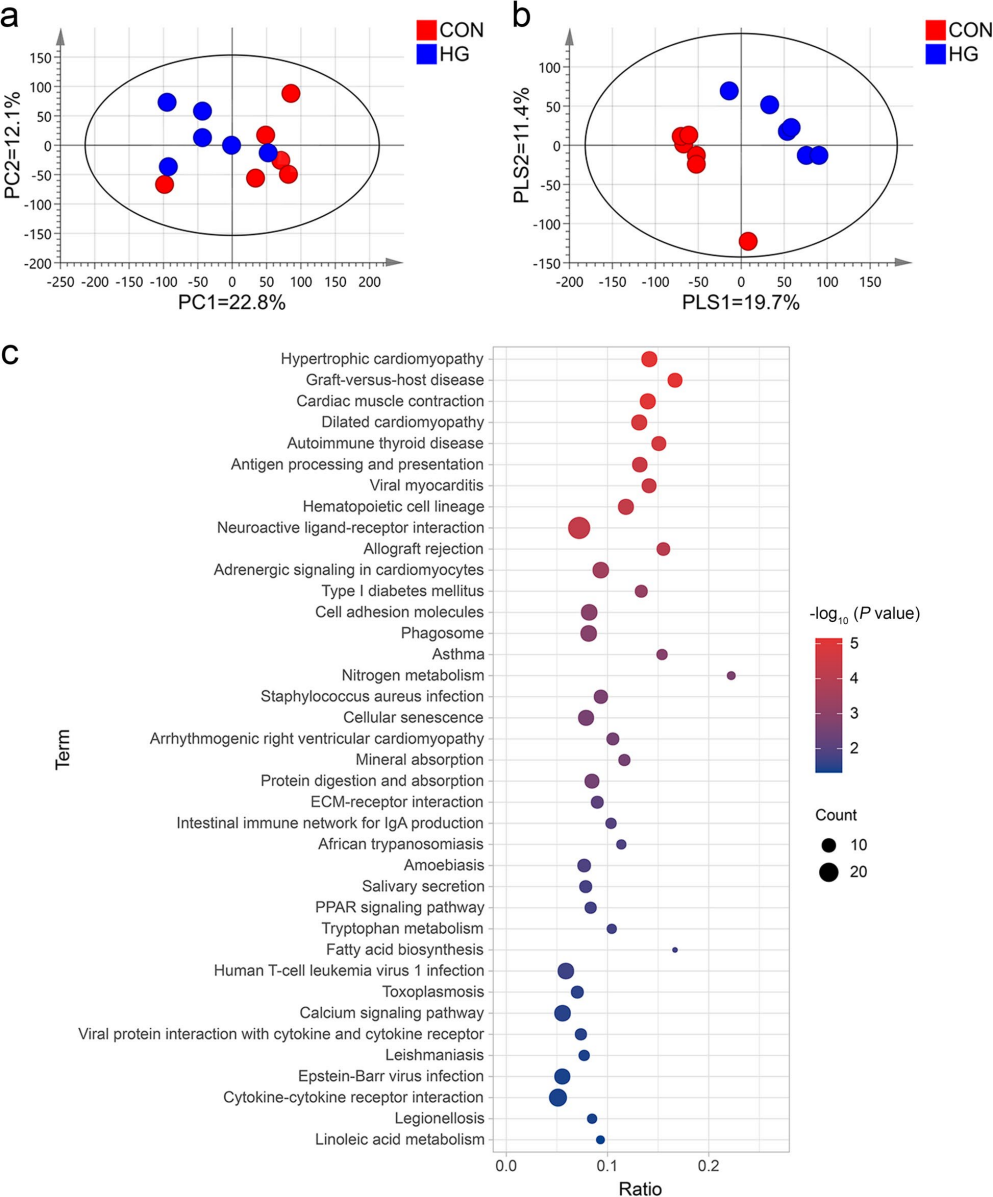
The principal component analysis (PCA) and the partial least squares discriminant analysis (PLS-DA) of the adipose tissue transcriptome for dairy cows in the conventional diet group (CON; 40% concentrate, *n* = 6) group and the high-grain diet group (HG; 60% concentrate, *n* = 6) group. **a** PCA score scatter plot; **b** PLS-DA score scatter plot. **c** Kyoto Encyclopedia of KEGG pathway enrichment analysis of differentially expressed genes (DEGs). The *X*-axis and *Y*-axis in (**c**) represent the pathway and the pathway enrichment ratio, respectively

The entire high-quality dataset of transcriptome sequencing of adipose tissue was used to perform the PCA to investigate the clustering of the samples. The PCA plot (Fig. 4a) shows that HG and CON gene expression profiles of adipose tissue seem to fall into distinct clusters. It is easy to see that PC1 and PC2 explained 22.8% and 12.1% of the total variation, respectively. The PLS-DA results showed that the samples of the CON and HG groups were divided into two clusters (PLS1 = 19.7% and PLS2 = 11.4%, Fig. 4b), which indicates significant differences between the two groups at the transcriptome level. Compared with the CON group, a total of 1061 DEGs (fold change ≥ 2 and FDR < 0.05) were identified from the 23,097 genes in the HG group, including 504 up-regulated genes and 557 down-regulated genes. Figure 4c shows 38 significantly changed pathways (*P* < 0.05). Among these, three pathways are related to lipid metabolism: fatty acid biosynthesis, linoleic acid metabolism, and the PPAR signalling pathway. A total of 13 DEGs were identified in these three pathways, 8 of which were significantly up-regulated and 5 were significantly down-regulated in the HG group compared with the CON group (Table 8). In the fatty acid synthesis pathway, HG feeding increased the gene expressions of *FASN*, *ACACA*, and *ACSBG2* (Fig. S4 in Additional file 1). In the linoleic acid metabolism pathway, the gene expressions of *CYP3A24*, *CYP2E1*, *HRAS-3*, and *PLA2G2D1* were up-regulated by feeding the cows HG diets (Fig. S5 in Additional file 1). In the PPAR signalling pathway, the cows on a HG diet had up-regulated gene expressions of *UCP1* and *ACSBG2* in adipose tissue, while the expression levels of *APO-A1*, *OLR1*, *APO-C3*, *SLC27A2*, and *ANGPTL4* were down-regulated (Fig. S6 in Additional file 1).

**Table 8:** Differentially expressed genes in abdominal adipose tissue due to high-grain diet feeding^1^

Pathway	Gene symbol^2^	FDR	Log_2_FC	Gene-ID
Fatty acid synthesis	*FASN*	< 0.001	1.375	24,950
	*ACACA*	< 0.001	1.011	23,708
	*ACSBG2*	0.037	1.683	9568
Linoleic acid metabolism	*PLA2G2D1*	< 0.001	2.239	2731
	*CYP3A24*	< 0.001	4.498	30,249
	*CYP2E1*	< 0.001	13.169	30,981
	*HRAS-3*	< 0.001	14.152	32,537
PPAR signalling pathway	*UCP1*	< 0.001	3.011	21,041
	*ACSBG2*	0.037	1.683	9568
	*APO-A1*	< 0.001	−1.230	18,573
	*OLR1*	0.009	−1.145	7294
	*APO-C3*	0.002	−1.003	18,572
	*SLC27A2*	0.047	−11.379	13,784
	*ANGPTL4*	< 0.001	−1.228	9509

^1^Results based on RNA sequencing, *n* = 6. The gene expressions were considered to be significantly altered when fold change ≥ 2 and FDR < 0.05

^2^*FASN F*atty acid synthetase, *ACACA* Acetyl-CoA carboxylase, *ACSBG2* Long-chain-fatty-acid–CoA ligase ACSBG2 isoform X1, *CYP3A24* Cytochrome P450 3A24, *CYP2E1* Cytochrome P450 2E1, *HRAS-3 *HRAS-like suppressor 3, *PLA2G2D1* Calcium-dependent phospholipase A2 PLA2G2D1 precursor, *UCP1* Mitochondrial brown fat uncoupling protein 1, *APO-A1* Apolipoprotein A-I preproprotein, *OLR1* Oxidized low-density lipoprotein receptor 1, *APO-C3* Apolipoprotein C-III precursor, *SLC27A2* Very long-chain acyl-CoA synthetase, *ANGPTL4* Angiopoietin-related protein 4 precursor

### qPCR validation of the RNA-seq

**Fig. 5:**
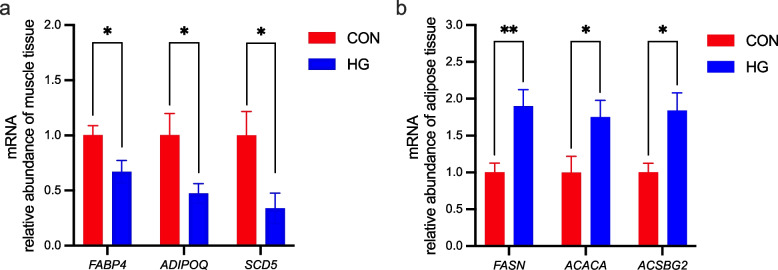
The mRNA expression abundance of genes in muscle and adipose tissue of dairy cows between the conventional diet group (CON) and the high-grain diet group (HG). **a** Muscle tissue; **b** adipose tissue. ^*^P < 0.05, ^**^P < 0.01

Quantitative real-time PCR was conducted to verify the transcriptome results. The expression patterns of *FASN*, *ACACA*, *ACSBG2*, *FABP4*, *ADIPOQ*, and *SCD5* were consistent with the transcriptome data. Briefly, in the HG group, the gene expressions of *FABP4*, *ADIPOQ*, and *SCD5* were down-regulated (*P* < 0.05) in the muscle tissue (Fig. 5a), while the gene expressions of *FASN*, *ACACA*, and *ACSBG2* were up-regulated (*P* < 0.05) in the adipose tissue (Fig. 5b).

## Discussion

SARA induced by increasing easily fermentable carbohydrates and reducing the fibre intake in the HG diet results in the accumulation of short-chain fatty acids in the rumen, as well as a reduction in chewing capacity, salivary buffer supply, and rumen motility [7]. In this study, a short-term SARA was induced using a model developed by Keunen et al. by feeding dairy cows with HG diet for 3 weeks [25, 38, 39]. Detection of rumen pH is a common method for the diagnosis of SARA, while the definitive rumen pH threshold for SARA is various in the literature. According to the ruminal microbial activity, the ruminal pH measurement and the integrity of the rumen epithelium, ruminal pH 5.5, 5.8, and 6.0 were used to diagnose SARA [5, 6, 40]. The rumen pH below 5.8 for more than 180 min/d will trigger an inflammatory response during SARA [25]. In the present study, the rumen pH of the cows in the HG group was below 5.8 which lasted for more than 3 h within a day, indicating that SARA has been successfully induced.

Milk fat content is a crucial index for measuring the performance of dairy cows. Our data demonstrated that feeding HG diet can affect the performance of dairy cows, which is manifested by the change of fat content, protein content, and fatty acid composition in milk. In this study, HG feeding reduced milk fat content and increased milk protein content but did not affect milk yield and other milk characteristics, which concurred with previous results [41]. Since the synthesis of milk fat is the result of the comprehensive metabolism of dairy cows after dietary intake, the decrease in milk fat content indicates that SARA causes abnormal fat metabolism in the body and further inhibits the synthesis of milk fat. Data from this research showed that DMI was not different between the two dietary treatments. Paradoxically, several researchers have reported that excessive grain feeding decreased the DMI [42, 43]. The effects of HG diets on DMI were inconsistent, and the variance in diet formula, the length of the experimental period, or the individual differences between the cows are responsible for this result.

Most short- and medium-chain fatty acids (C ≤ 16) in milk come from de novo synthesis in mammary tissue [9]. The analysis of milk fatty acid composition in our study showed that adding high amounts of grain to a cow’s diet increases the proportion of short- and medium-chain fatty acids (C10:0, C11:0, C12:0, and C16:1 *cis*9) in its milk. These changes suggest shifts in the de novo synthesis of milk fat [44]. Furthermore, we also found HG feeding decreased the proportion of long-chain fatty acids (C17:0 and C18:0) in the milk. It is well-accepted that most long-chain fatty acids (C > 16) in milk are absorbed from the blood, and there is a strong link between the distribution of fatty acids in blood and milk [45]. In our study, data on long-chain fatty acids in plasma and blood further supported this view, showing that the trend of C > 16 in plasma of cows in the HG group was consistent with that in milk. Consistent with previous reports, HG feeding had no significant effect on the SFA, UFA, MUFA, and PUFA concentrations in the milk [46, 47].

The concentration of plasma TG is a commonly used biomarker for lipid metabolism [48]. In the present study, switching to a HG diet tended to decrease the plasma TG concentration, which is associated with the decreased lipolysis and ruminal biohydrogenation of dietary fatty acids under the low ruminal pH [49] or the insufficient supply of endogenous fatty acids from liver and adipose tissue [24, 50].

Grain-based SARA increases ruminal and peripheral bacterial endotoxin concentrations [51]. Rumen endotoxin can be released into the peripheral circulation causing systemic inflammation, and its concentration is negatively correlated with plasma TCHO concentration [52, 53]. In the present study, the TCHO level was reduced in cows fed a HG diet. Although our study did not assess rumen endotoxin concentrations, the low rumen pH in the HG group will result the death of a large number of Gram-negative bacteria in the rumen and the release of endotoxin should be expected. At the same time, LDL, as an essential lipoprotein primarily responsible for cholesterol transport [54], had a correspondingly reduced concentration in the plasma of the cows on the HG diet in the present study. Since TG and TCHO are the two main lipids in plasma, feeding HG diet changes their plasma concentrations, revealing that HG diet changes lipid profiles in blood of dairy cows.

Another key finding of our study was that excessive grain feeding significantly altered the plasma fatty acid composition of dairy cows. The HG group had higher proportions of C4, C6, C17:1 *trans*10, and C ≤ 16 and lower proportions of C16:1 *cis*9, C18:2 *cis*9,12, C20:3 *cis*8,11,14, and C > 16 compared with the CON group. The PUFA status of ruminants is particularly precarious due to the hydrogenation of UFAs by rumen microbes [55]. PUFAs in blood originate from the uptake of pre-formed fatty acids from the rumen [56]. Our results showed that HG feeding decreases the blood PUFA proportion in dairy cattle, which suggests the alterations in rumen biohydrogenation. In addition, levels of C4 and C6 fatty acids in milk and plasma in this study are likely lower than in previous reports. This might be caused by the loss of short fatty acids in the process of fatty acid methyl ester production, as we did not use correction factors to correct this result.

Muscle is a key regulatory tissue that helps the body respond to changes in energy and lipid metabolism during lactation [57]. Studies on the effects of diet on muscle tissue have focused on beef cows [58, 59]; however, the literature on dairy cows is scarce. Different dietary regimes have been shown to influence the fatty acid profile of beef [60]. Scollan et al. [61] reported that beef cows fed a forage-based diet had higher concentrations of n-3 PUFAs in their muscle tissue than beef cows fed a grain-based diet. Nevertheless, due to the differences of breed and experiment period, we did not find significant effects of HG diet on fatty acid composition in muscle tissue of dairy cows in this study. However, transcriptomic data from muscle tissue indicate that switching to a HG diet altered the UFA biosynthesis pathway by down-regulating the gene expressions of *SCD5* and *ELOVL6*.

In current study, gene expression of *FABP4* and *ADIPOQ* in the muscle of HG group was lower than the CON group. *FABP4* is associated with the absorption, transport, and metabolism of fatty acids and mediates the transfer of fatty acids during lipolysis [62]. *ADIPOQ* is one of the most abundant circulating adipokines and is negatively correlated with fat mass [63]. We found that HG feeding tended to raise the TG concentration in the muscle tissue of dairy cows. At the transcription level, the lower expression levels of *FABP4* and *ADIPOQ* in the HG group is suggestive of decreased fatty acid transport capacity during lipolysis and increased fat mass in muscle tissue.

In line with our hypothesis, the fatty acid composition of adipose tissue was affected by HG feeding. Overall, the cows on the HG diet showed a tendency for a higher proportion of C16:1 *cis*9 and a lower proportion of C18:1 *cis*9 in their adipose tissue compared with cows on the CON diet. One previous study in humans clearly shows that MUFAs regulate lipid metabolism through modifications in the composition of cell membranes [64]; however, the effects of MUFAs on ruminants need to be further studied. In the present study, the increased TG concentration and activated fatty acid synthesis pathways in the adipose tissue of cows on a HG diet revealed that HG intake enhances lipid synthesis capacity in the adipose tissue of dairy cows. *FASN*, *ACACA*, and *ACSBG2* are genes involved mainly in the fatty acid synthesis pathway, which encodes key enzymes linked to fatty acid synthesis [9]. In the present study, the up-regulated expressions of *FASN*, *ACACA*, and *ACSBG2* in the adipose tissue of cows on a HG diet provide transcriptional evidence for the theory of Baldwin et al. [65], which states that the activity of enzymes involved in fatty acid synthesis in adipose tissue increases when diets contain high amounts of grain. Additionally, it has been noted that *FASN* also participates in the regulation of TG synthesis [66]. The results of this study showing that the up regulated gene expression of *FASN* is accompanied by an increase in TG content could support this view. Furthermore, HG diets transfer nutrients from milk fat synthesis to body fat synthesis [67], which is also confirmed by our research at transcription level. In this study, the linoleic acid metabolism pathway in adipose tissue was also activated by HG feeding. The higher gene expression levels of *CYP3A24*, *CYP2E1*, *HRAS-3*, and *PLA2G2D1* will lead to the conversion of more lecithin to linoleic acid and subsequently to epoxyoctadecanoic acid. However, linoleate is an important component of lipoproteins and is involved in the transport of lipids [68]. Thus, our results suggest that feeding HG diet can reduce the lipid transport capacity in the adipose tissue of dairy cows by regulating gene expression related to linoleic acid metabolism.

The PPAR signalling pathway consists of interrelated genes that encode transcription factors, enzymes, and downstream targets that coordinately act to regulate lipid uptake, synthesis, and transport [69]. Interestingly, in the current study, the DEGs of both muscle and adipose tissue marked this pathway, indicating that HG feeding affects the lipid metabolism of muscle and adipose tissue by regulating gene expression in the PPAR pathway.

## Conclusion

In conclusion, a relatively short-term HG feeding induced SARA, significantly decreased the milk fat content, and altered the milk fatty acid composition of dairy cows. The HG diet reduced the TG content in the blood and changed the fatty acid composition of the cows’ plasma. In muscle tissue, HG feeding tended to increase TG concentration but did not affect fatty acid composition. However, in adipose tissue, the HG diet significantly increased the TG concentration and changed the fatty acid composition. Finally, transcriptome analysis of muscle and adipose tissue showed that HG diets could increase lipid synthesis and decrease lipid transport.

## Supplementary Information

**Additional file 1: Table S1.** Primer sequences are used for real-time PCR analysis. **Fig. S1.** Regulation of lipolysis in adipocytes signaling KEGG pathway in muscle tissue. **Fig. S2.** Biosynthesis of unsaturated fatty acids signaling KEGG pathway in muscle tissue. **Fig. S3.** PPAR signaling KEGG pathway in muscle tissue. **Fig. S4.** Fatty acid biosynthesis signaling KEGG pathway in adipose tissue. **Fig. S5.** Linoleic acid metabolism signaling KEGG pathway in adipose tissue. **Fig. S6.** PPAR signaling KEGG pathway in adipose tissue.

## Data Availability

All data generated or analyzed during this study are available from the corresponding author on reasonable request.

## Abbreviations

BW: Body weight
CP: Crude protein
DEGs: Differentially expressed genes
DM: Dry matter
DMI: Dry matter intake
FAMEs: Fatty acid methyl esters
FC: Fold change
FDR: False discovery rate
F/G: Feed/gain ratio
FPKM: Fragments per kilobase per million mapped fragments
GO: Gene ontology
HG: High-grain
KEGG: Kyoto encyclopedia of genes and genomes
NDF: Neutral detergent fiber
NE: Net energy
NFC: Non-fibrous carbohydrate
PUFAs: Polyunsaturated fatty acids
SARA: Subacute rumen acidosis
TG: Triacylglycerol
