# Supplementary material for: Effects of high-grain diet feeding on fatty acid profiles in milk, blood, muscle, and adipose tissue, and transcriptional expression of lipid-related genes in muscle and adipose tissue of dairy cows
Source: J Anim Sci Biotechnol. 2023 Apr 8;14:41. doi: 10.1186/s40104-023-00847-y (PMC10082502; doi:10.1186/s40104-023-00847-y)
Supplement: Supplementary file 1 — Additional file 1: Table S1. Primer sequences are used for real-time PCR analysis. Fig. S1. Regulation of lipolysis in adipocytes signaling KEGG pathway in muscle tissue. Fig. S2. Biosynthesis of unsaturated fatty acids signaling KEGG pathway in muscle tissue. Fig. S3. PPAR signaling KEGG pathway in muscle tissue. Fig. S4. Fatty acid biosynthesis signaling KEGG pathway in adipose tissue. Fig. S5. Linoleic acid metabolism signaling KEGG pathway in adipose tissue. Fig. S6. PPAR signaling KEGG pathway in adipose tissue. [file 40104_2023_847_MOESM1_ESM.docx]

| **Table S1** Primer sequences are used for real-time PCR analysis | | | |
| --- | --- | --- | --- |
| **Accession ID** | **Gene** | **Primer sequence (5’→3’)** | **Amplicon size, bp** |
| NM_001012669 | *FASN* | F: ACCTCGTGAAGGCTGTGACTCA | 92 |
|  |  | R: TGAGTCGAGGCCAAGGTCTGAA |  |
| NM_174314 | *ACACA* | F: CATCTTGTCCGAAACGTCGAT | 101 |
|  |  | R: CCCTTCGAACATACACCTCCA |  |
| XM_024994960 | *ACSBG2* | F: TTTTGTGGGCAACCAATATTGG | 113 |
|  |  | R: CTTGATTTTGGTGAACACGAGT |  |
| NM_174314 | *FABP4* | F: CCAAACCCACTTTGATCATCGA | 147 |
|  |  | R: TTACGATGCTCTTGACTTTCCT |  |
| NM_174742 | *ADIPOQ* | F: CTTAAGGAAGTAGCTTGAGCCT | 211 |
|  |  | R: TGAAGCAATCACCACAATCAAG |  |
| NM_001076945 | *SCD5* | F: TCATGGCCTTTGTTACTACACT | 203 |
|  |  | R: ATTAAATCACATGTCCGCGTTC |  |
| NM_001034034.2 | *GAPDH* | F: GGGTCATCATCTCTGCACCT | 180 |
|  |  | R: GGTCATAAGTCCCTCCACGA |  |

**
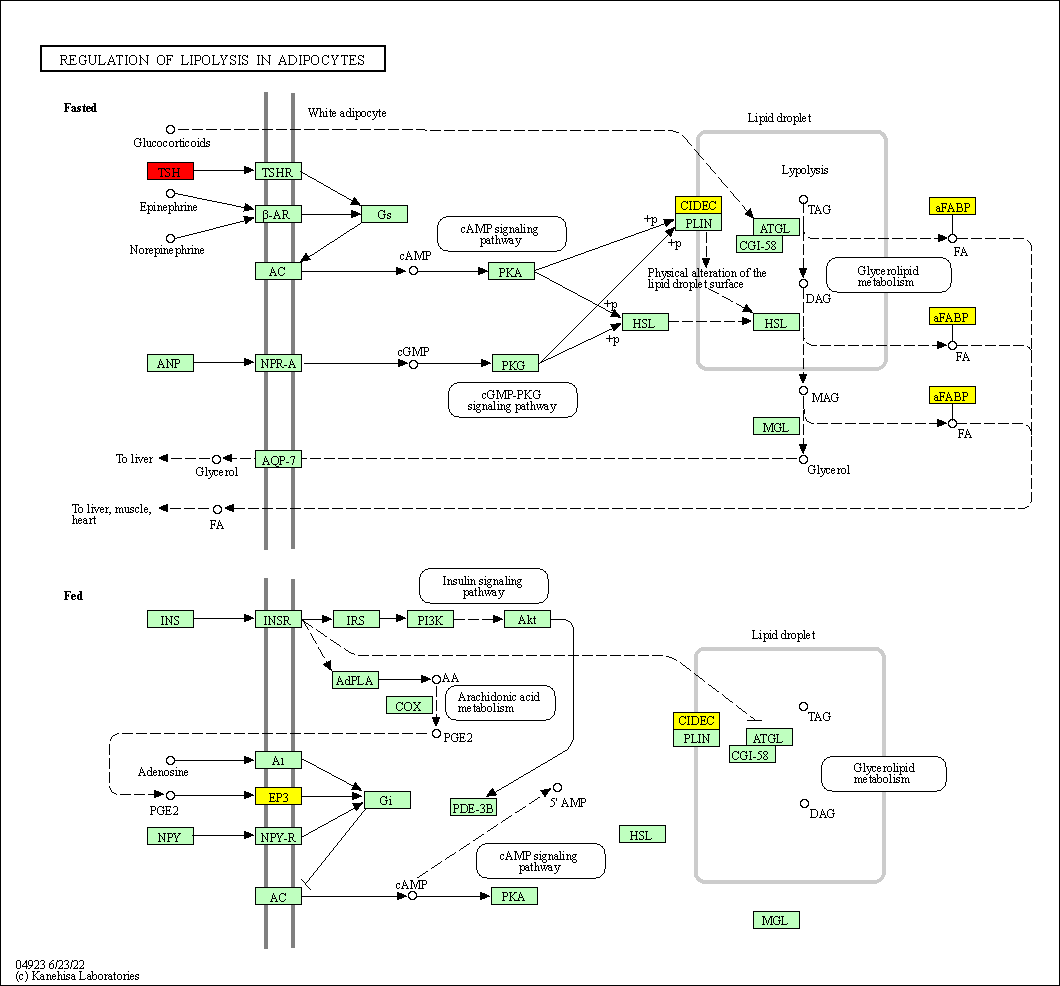
**

**Fig. S1** Regulation of lipolysis in adipocytes signaling KEGG pathway in muscle tissue. Yellow shade denotes down-regulation while red shade denotes up-regulation of genes in the high-grain group

**
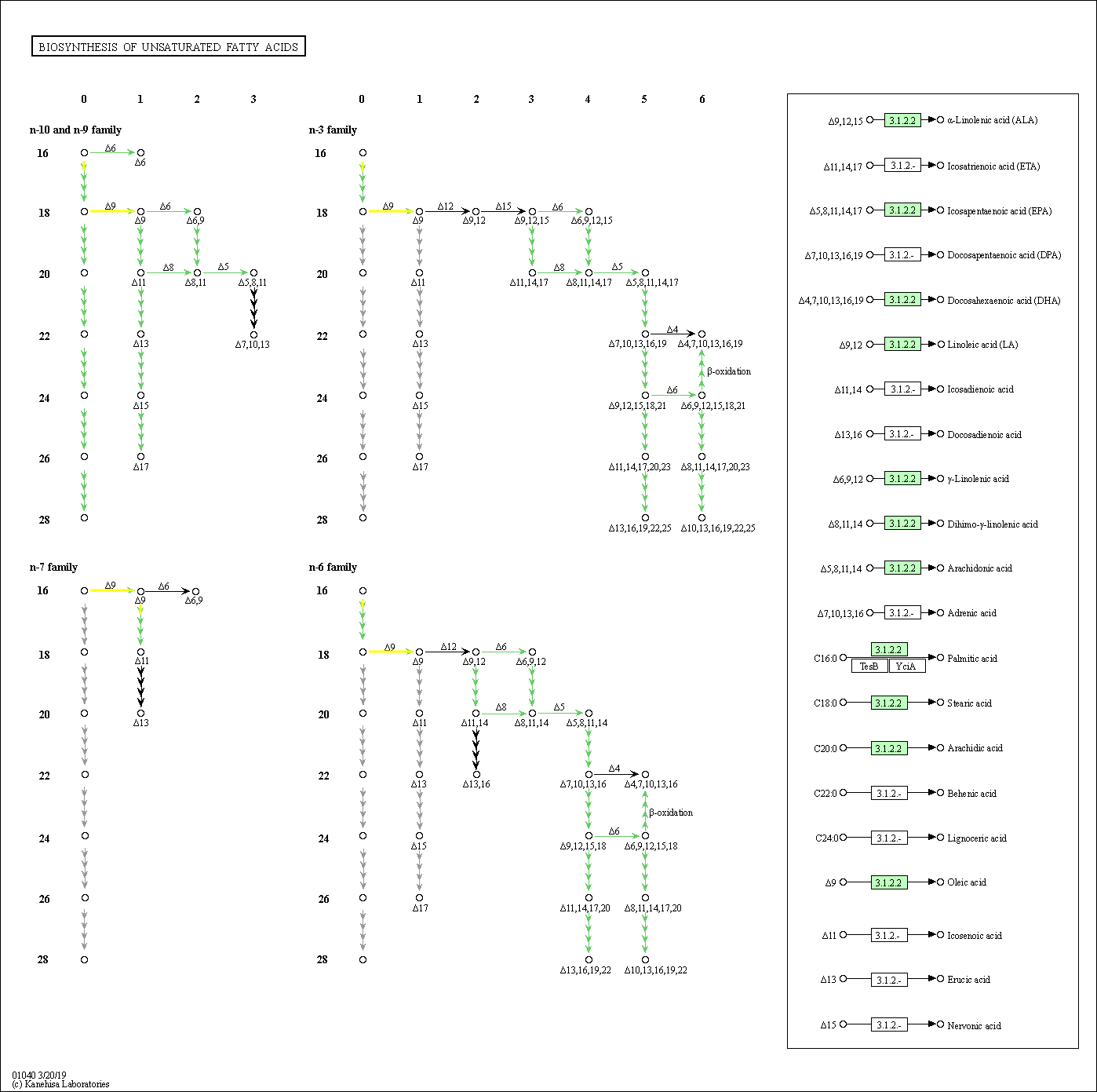
**

**Fig. S2** Biosynthesis of unsaturated fatty acids signaling KEGG pathway in muscle tissue. Yellow shade denotes down-regulation while red shade denotes up-regulation of genes in the high-grain group

**
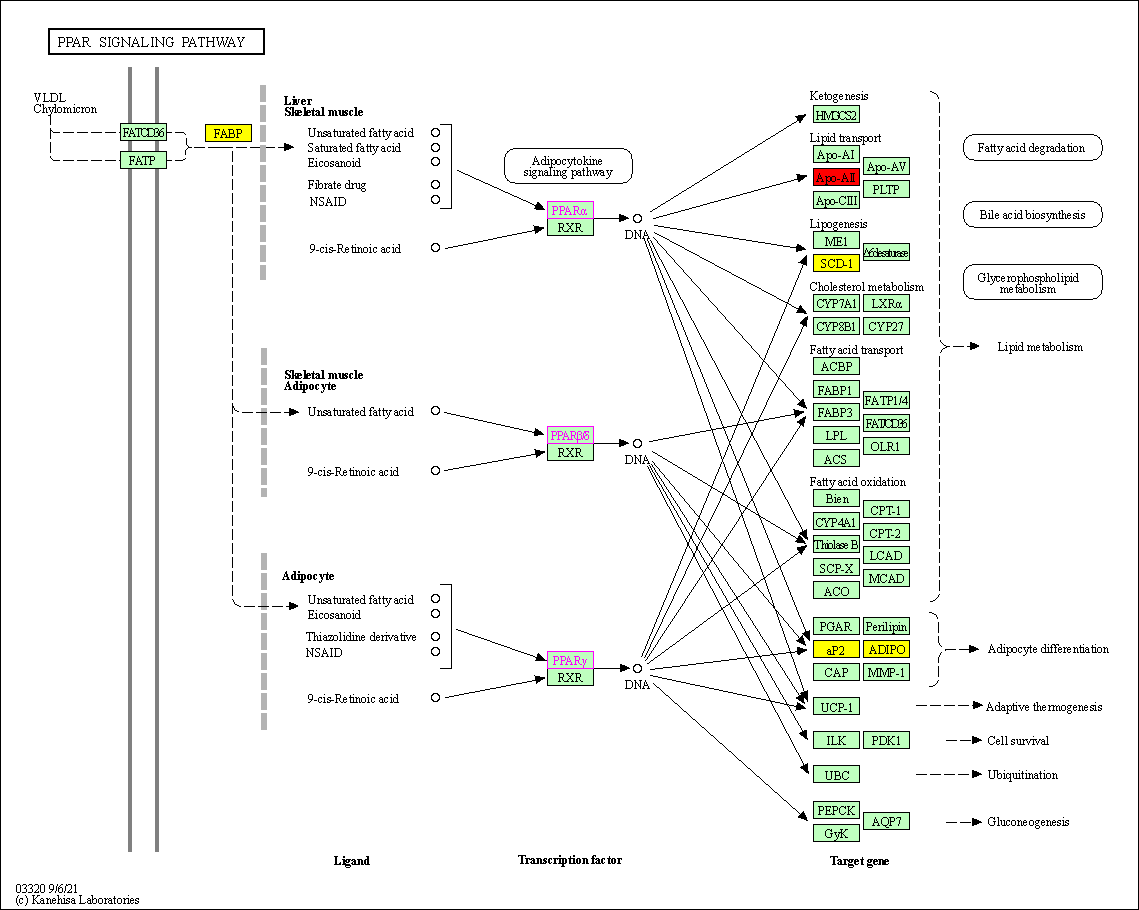
**

**Fig. S3** PPAR signaling KEGG pathway in muscle tissue. Yellow shade denotes down-regulation while red shade denotes up-regulation of genes in the high-grain group

**
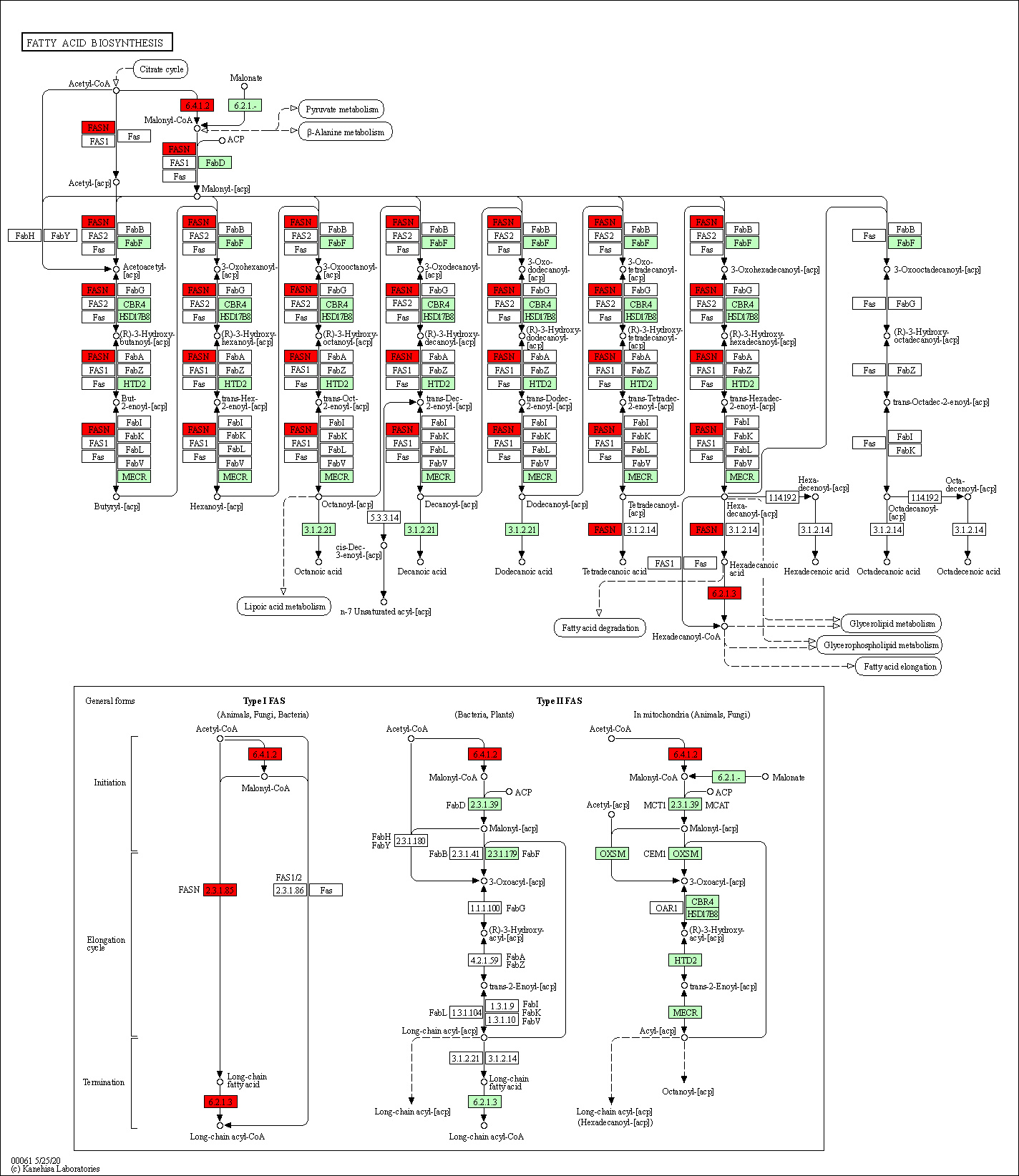
**

**Fig. S4** Fatty acid biosynthesis signaling KEGG pathway in adipose tissue. Yellow shade denotes down-regulation while red shade denotes up-regulation of genes in the high-grain group

**
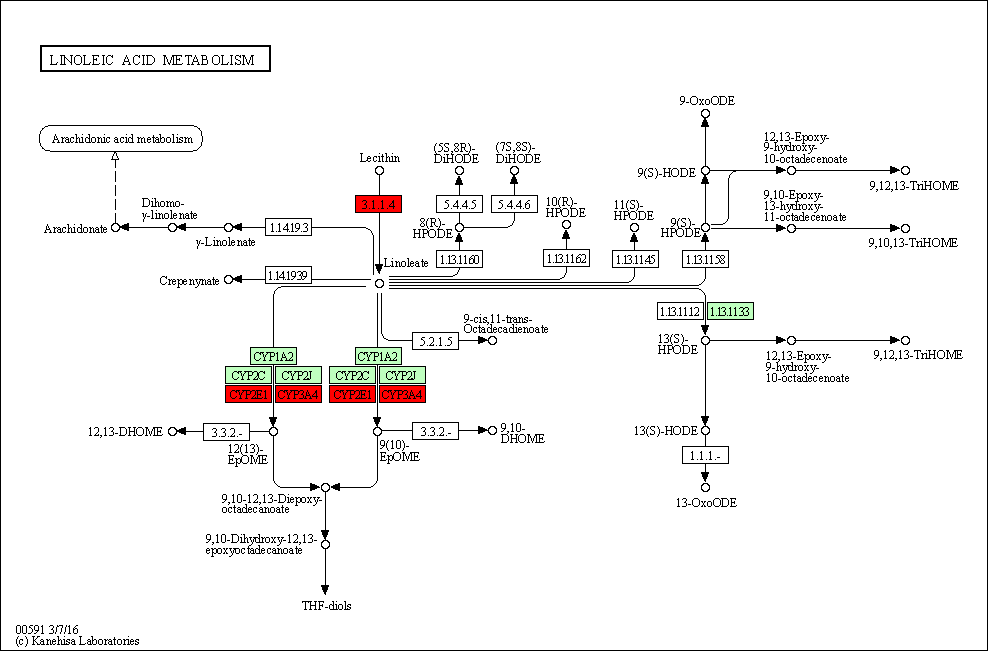
**

**Fig. S5** Linoleic acid metabolism signaling KEGG pathway in adipose tissue. Yellow shade denotes down-regulation while red shade denotes up-regulation of genes in the high-grain group

**
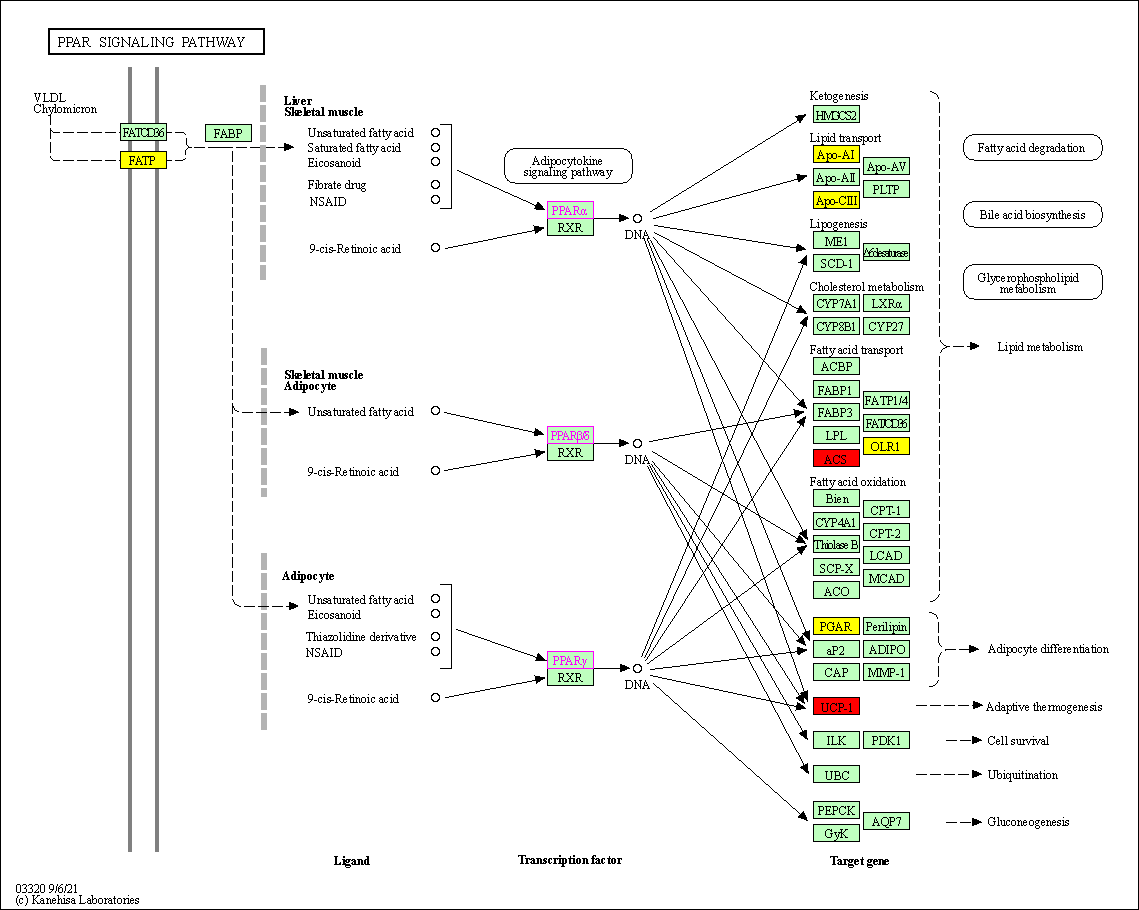
**

**Fig. S6** PPAR signaling KEGG pathway in adipose tissue. Yellow shade denotes down-regulation while red shade denotes up-regulation of genes in the high-grain group
